# Estimation of salivary cortisol level in post-menopausal women with psychosomatic disorders

**DOI:** 10.4314/ahs.v18i2.7

**Published:** 2018-06

**Authors:** Rao Kumuda, Kumari Suchetha, G Babu Subhas, A Shetty Urvashi, Ullal Harshini

**Affiliations:** 1 Department of Oral Medicine and Radiology, A B Shetty Memorial Institute of Dental Sciences, NITTE deemed to be University, Deralakatte, Mangalore; 2 Department of Biochemistry, K.S. Hegde medical academy, NITTE deemed to be University, Deralakatte, Mangalore; 3 Department of Oral Medicine and Radiology, A B Shetty Memorial Institute of Dental Sciences, NITTE deemed to be University, Deralakatte, Mangalore; 4 Department of Oral Pathology and Microbiology, A B Shetty Memorial Institute of Dental Sciences, NITTE deemed to be University, Deralakatte, Mangalore; 5 Central Research Laboratory, K. S. Hegde Medical Academy, NITTE deemed to be University, Deralakatte, Mangalore

**Keywords:** Post-menopausal women, psychosomatic disorder, head and neck, salivary cortisol, biomarker

## Abstract

**Background:**

Stress is an undesirable or health threatening response of the body, which is brought on by deleterious external influences (stressors). Objective measurement of psychosocial stress helps in assessment of pivotal role of stress in precipitation of multitude of health problems and a solution to the same. Salivary biomarkers are suggested to provide a reliable and non-invasive method for the estimation of these general health problems. Salivary cortisol is such biomarker used as tool in the examination of human physiological stress response. Post-menopausal women show an increase in stress levels and hence suffer with multiple health related problems. Hence the present study aimed to estimate salivary cortisol levels in post-menopausal women with clinically diagnosed psychosomatic disorder/disorders of the head and neck region, so as to establish salivary cortisol as a biochemical indicator of stress.

**Methods:**

Thorough intra-oral and extra-oral examination was performed to check for the presence of psychosomatic disorder of head and neck. Unstimulated saliva was collected from 100 post-menopausal women with and 100 without clinically diagnosed psychosomatic disorder/disorders through ‘Spit Technique’. Salivary cortisol was estimated using ELISA method.

**Results:**

The results were statistically significant as they showed that the salivary cortisol was in higher levels in post- menopausal women with clinically diagnosed psychosomatic disorder/disorders.

**Conclusion:**

The geriatric patients feel that they have very little skills or resources to deal with the high levels of stress that they are experiencing and hence suffer from lack of self-worth. The results of this study recommend that stress evaluation should be done on a regular basis for all post- menopausal women. For individuals who do not reveal their psychological distress, salivary analysis of cortisol may be used as an aid to diagnose their situation in conjunction with clinical diagnosis.

## Introduction

Psychiatric disorders are conventionally classified into organic group and functional group. In organic disorders like in dementia or delirium, a known physical etiology can be established but in functional disorders such as schizophrenia which comprise of a large majority of psychiatric illnesses, no particular physical factors are present.^1^ The first edition of “Diagnostic and Statistical Manual of Mental Disorder” (DSM-1) included the “Psychosomatic Disorders” in 1952 and DSM-II published it as “Psycho Physiological Autonomic and Visceral Disorder” in 1968. In 1980's DSM-III has renamed it has a “Psychological Factor Affecting the Physical Conditions”.^2^ Psychosomatic Disorders is defined by DSM-II, (1968) as psychosomatic symptoms that are caused by emotional factors and involve a single organ system, usually under autonomic nervous innervations.^2^ Stress is an undesirable or health threatening response of the body, brought about by harmful external influences called stressors.^3^

The hypothalamic-pituitary-adrenal (HPA) axis is a major homeostatic system that maintains an organism's equilibrium within its environment^4^. The HPA axis is the primary mammalian system of stress response, and the endpoint of HPA-axis activation is the release of the glucocorticoid cortisol. The principal role of cortisol during the stress response is to restrain the effectors of the stress response.^5^ The neural circuitry involved in fear and anxiety is modulated by brain neurotransmitters and other chemical mediators including hormones. A relevant hormonal system is the hypothalamo-pituitary-adrenal axis (HPA), which regulates cortisol secretion.^6^ Several studies have provided evidence of an association between HPA dysregulation and psychiatric symptoms.^7^,^8^ Depressed adults show increased total cortisol secretion, and is characteristic of HPA-axis hyperactivity.^9^

Very few studies have been undertaken to estimate the levels of cortisol in saliva. This study is one of its kinds as the clinical manifestations of psychosomatic disorders secondary to stress has been taken into consideration. Oral Lichen planus [OLP], Apthous ulcers, Burning mouth syndrome [BMS], Tempero-Mandibular Disorders [TMD'S], Myofacial Pain Dysfunction Syndrome [MPDS], Atypical facial pain, Glossopyrosis, Dysgeusia, Xerostomia, Attrition, Halitosis are the most common oral lesions and symptoms secondary to psychosomatic disorders that affect the head and neck, and have been associated with stress. Numerous studies have proved that the etiological factors causing these disorders are multifactorial and are stress related.^10^

Salivary cortisol is such a biomarker used as a tool in the examination of chronic physiological stress.^11^,^12^–^14^ Salivary biomarkers are suggested to provide a reliable, non-invasive and objective measurement of chronic psychosocial stress and in turn helps in the assessment of pivotal role of stress in causation or precipitation of multitude of health problems. The present study aims to estimate and clinically establish salivary cortisol as a biomarker of stress by estimation of their levels among post-menopausal women with psychosomatic disorders.

## Materials and methods

The study was conducted in the time period of 2014 to 2016. Informed consent was obtained from the subjects included in the study. Detailed case history was recorded along with thorough examination of the oral cavity. Presence of psychosomatic disorder was recorded in case group with thorough intra-oral and extra-oral examination. To avoid bias both principal investigator and co-investigator examined the study subjects. Subjects who had positive history to stress and with stress related disorders enlisted in the inclusion criteria were included in the study group.

100 women subjects above 45 years of age, with minimum 2 years post-menopausal history, who did not have any oral or systemic diseases/pathologies or who were not on any medication were included in the study. The study group consisted of 100 women subjects above 45 years of age, with minimum 2 years post-menopausal history with any one or more of the psychosomatic disorders like Tempero-Mandibular Disorders [TMD'S], Myofacial Pain Dysfunction Syndrome [MPDS], Apthous stomatitis, Atypical facial pain, Oral lichen planus(OLP), Burning mouth syndrome [BMS]/ Glossopyrosis, Dysgeusia, Xerostomia, Attrition, Halitosis as control group. Subjects with a history of any long term psychiatric disorders and/or psychiatry patients were excluded from the study.

### Saliva collection

Subjects were instructed not to eat, drink or smoke 1 hour prior to sample collection They were then seated on the dental chair with their head tilted forward and asked not to speak or swallow any saliva. The subjects were then instructed to spit into a sterile graduated container every minute for 8–10 minutes. Accumulated saliva was collected by ‘spit method’. 2 ml of collected saliva was stored at a temperature of −200 C in plastic vials and analysis was carried out within 24 hours. Unstimulated saliva of 200 volunteers was collected.The salivary sample represented whole mouth fluid (saliva from major and minor salivary glands and gingival crevicular fluid). Saliva collection was limited to the hours between 9:00 and 11:00 to minimise diurnal variation. The collected sample was centrifuged at 3000 rpm for 10 minutes and the supernatant collected was stored at −20°C.

Analysis of salivary Cortisol was done using ELISA method at the NITTE University Central Research Cell at K S Hegde Medical Academy.

### Measurement of salivary cortisol by ELISA method:

(LDN Labor Diagnostika Nord GmbH & Co. KG)

### Principle

The Cortisol free in Saliva ELISA Kit is a solid phase Enzyme-Linked Immuno-Sorbent Assay (ELISA), based on the principle of competitive binding. The microtiter wells are coated with a polyclonal rabbit antibody directed against the cortisol molecule. Endogenous cortisol of a patient sample competes with a cortisol-horseradish peroxidase conjugate for binding to the coated antibody. After incubation the unbound conjugate is washed off. The amount of bound peroxidase conjugate is inversely proportional to the concentration of cortisol in the sample. After addition of the substrate solution, the intensity of colour developed is inversely proportional to the concentration of cortisol in the patient sample.

### Statistical analysis

The data collected was entered into Microsoft excel spread sheet and analysed using IBM SPSS Statistics, Version 22(Armonk, NY: IBM Corp). Descriptive data were presented in the form of mean and standard deviation. Comparison of duration and age between the study groups was analysed using Independent sample t test. Pearson's correlation test was used to test the correlation between the groups. P value < 0.05 was considered as statistically significant.

## Results

The present study was conducted with an aim to evaluate salivary cortisol levels as biomarker of stress in post-menopausal women. The presence of psychosomatic disorders of head and neck among them indicated the manifestation of chronic stress. Hence 100 such subjects were included under study group. The control group consisted of 100 post-menopausal women without any psychosomatic disorder.

### Comparison of age and duration between the study groups was analysed using Independent sample t test.

**Graph 1:**
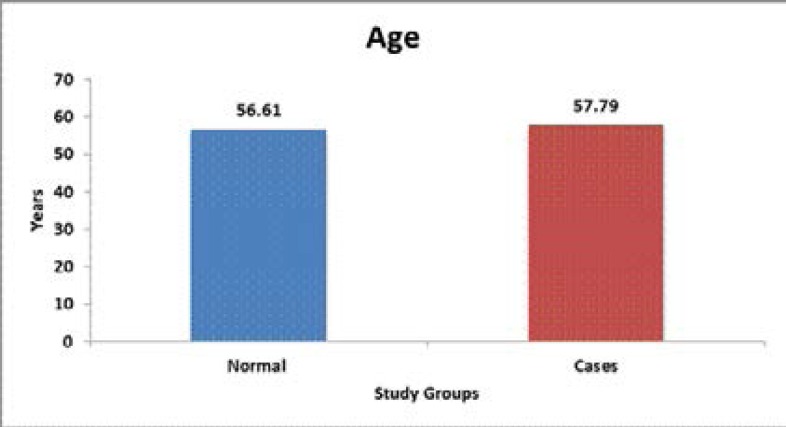
Comparison of age between the study groups

In the control group the mean age was 56.61 years and among the study group the mean age was 57.79 years. (Table 1, Graph 1). But the correlation of salivary cortisol with respect to age was statistically non-significant (Table 1).

**Table 1:** Comparison of duration and age between the groups

	Group	N	Mean	SD	Mean difference (95% CI)	t	df	p-value
**Duration**	**Normal**	100	5.50	3.58	−0.98 (−1.91, −0.05)	−2.07	198	0.04*
	**Cases**	100	6.48	3.08				
**Age**	**Normal**	100	56.61	6.69	−1.18 (−2.99, 0.63)	−1.29	198	0.20(NS)
	**Cases**	100	57.79	6.30				

Independent sample t test

P<0.05 statistically signficant, p>0.05 non significant, NS

**Graph 2:**
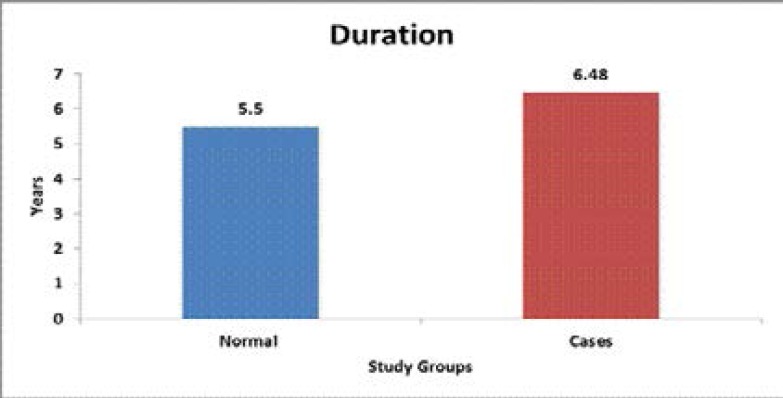
Comparison of duration of menopause between the study groups

The period of menopause ranged from 2 to 20 years among the control group and 2 to 15 years in the study group. The results were statistically significant as they showed higher levels of salivary cortisol with increased duration post-menopause (Table 1, Graph 2). The levels of salivary cortisol are plotted on scattered chart to show the distribution (Scatter chart 1).

**Figure:**
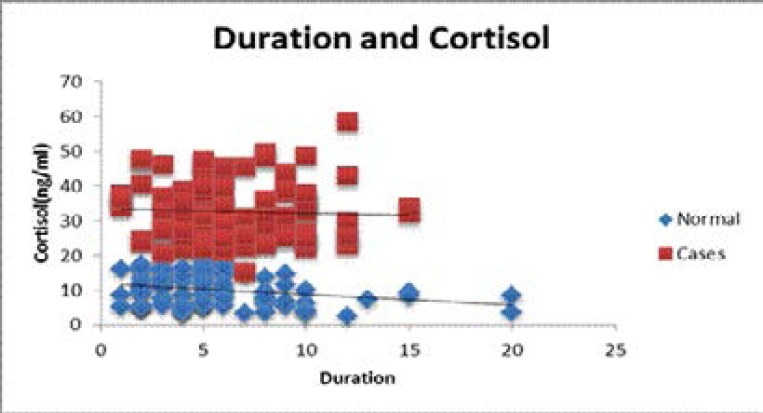
Scatter chart 1:

The mean salivary cortisol levels in control group and study group was found to be 10.24 ng/ml, and 32.73 ng/ml respectively. When the mean values were compared between the groups the values were statistically highly significant (Table 2, Graph 3)

**Table 2:** comparison of Cortisol between the study groups

	Group	N	Mean	SD	Mean difference (95% CI)	t	df	p-value
**Cortisol**	**Normal**	100	10.24	4.54	−22.49 (−24.36, −20.63)	−23.81	153.5	<0.001*
	**Cases**	100	32.73	8.29				

Independent sample t test

P<0.05 statistically signficant, p>0.05 non significant, NS

**Graph 3:**
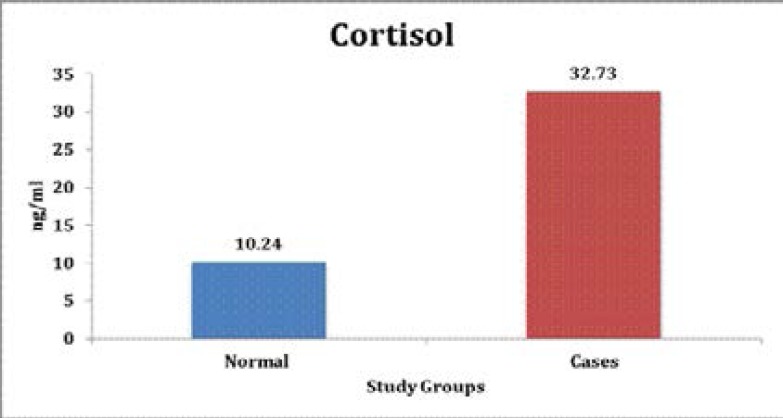
Comparison of salivary Cortisol between the study groups

Pearson's correlation test was used for Correlation between the study parameters in each study group. P value < 0.05 was considered as statistically significant (Table 3). Assessment of salivary cortisol was done among various psychosomatic disorders with mean values and standard deviation for each psychosomatic disorders that affect the head and neck, enlisted in the inclusion criteria. Mean value of salivary cortisol level was seen to be highest among women with Apthous stomatitis (37.97 ng/ml)and least among women who experienced halitosis (23.33 ng/ml) (Table 4)

**Table 3:** Correlation between the study parameters in each study group

Group			Age	Cortisol
**Normal**	**Duration**	**r**	0.56	−0.25
		**p-value**	<0.001*	0.01*
	**Cortisol (ng/ml)**	**r**	−0.03	1
		**p-value**	0.74(NS)	
	**Duration**	**r**	0.60	−0.05
		**p-value**	<0.001*	0.63(NS)
	**Cortisol (ng/ml)**	**r**	−0.07	1
		**p-value**	0.51(NS)	

Pearsons correlation test

P<0.05 statistically signficant, p>0.05 non significant, NS

**Table 4:** Correlation of salivary cortisol with various psychosomatic disorders

Condition		Cortisol (ng/ml)	
	N	Mean	SD
Tempero-Mandibular Disorders [TMD'S]	15	33.10	6.60
Myofacial Pain Dysfunction Syndrome [MPDS]	14	30.28	9.00
Apthous stomatitis,	8	37.97	3.03
Atypical facial pain	4	37.65	7.24
Oral lichen planus(OLP),	13	32.47	11.37
Burning mouth syndrome [BMS]/ Glossopyrosis	1	36.18	---.
Dysgeusia,	1	28.96	---.
Xerostomia,	3	25.33	12.07
Attrition,	2	34.26	16.13
Halitosis	1	23.33	---.
Multiple	38	32.72	7.58
TOTAL	100	32.73	8.29

## Discussion

Stress and factors leading to stress are a multi-dimensional construct.^15^ Role of psychosocial factors including stress in the changes of human body is one of most widely researched area of interest by psycho-physiologists. The role of salivary cortisol as a meaningful and reliable stress marker indicative of HPA axis function has been recognized since many years.^16^

Following the above studies, associations of oral lesions and cortisol have been consistently found and tried to be established. Studies which resulted in establishing a link between stress and increased levels of salivary cortisol have also been conducted as seen in studies by Jessop et al^6^, Schedlowski et al,^7^ Pomerleau et al,^8^ Rohleder N et al^13^ and Nater et al^14^. But studies on post-menopausal women with psychosomatic disorders for evaluation of salivary cortisol as biomarkers of stress have not been conducted. We have tried to establish the link between stress and psychosomatic disorders that affect the head and neck among the post-menopausal women by estimation of salivary cortisol.

Role of psychosocial factors like stress in the changes on human body is one of most widely researched area of interest by psycho-physiologists. The earlier studies were planned to evaluate the usefulness of hormone cortisol in saliva as a biomarker of stress among post-menopausal women; which usually results in extensive damage to the physical and mental wellbeing of an individual and may cause several pathologies. The role of cortisol in chronic stress has been identified in various serum and salivary studies as mentioned before. Hence this study aimed to correlate increase in salivary cortisol levels to the presence of psychosomatic disorders in the head and neck.

Numerous other studies where verbal and/or self-reporting questionnaires were used alone or in combination in the evaluation of stress have been conducted by Norozi et al,^17^ Potdar N et al,^18^ Nosek M et al^19^ which have provided highly inconsistent results requiring further research; probably owing to obvious alteration in the patient's mood and attitude. Usually individuals suffering from stress and related problems have a tendency to either deny or exaggerate the real condition; which leads to bias in the study and confounds with the results. This may be one of the main reasons why many previous studies conducted to find out the role of psychosocial factors in the manifestations of oral pathologies have reported contradictory results.^20^

In our study, the mean salivary cortisol levels in control group and study group was found to be 10.24 ng/ml, and 32.73 ng/ml respectively. When the mean values were compared between the groups, the values were highly statistically significant which suggested a higher stress levels among the study group.

Numerous studies in the past like conducted by Vineetha M et al,^20^ have tried to associate stress and oral lesions, but not many have showed the prevalence of different psychosomatic disorders affecting the head and neck among stressed post-menopausal women. In this study we have tried to associate salivary cortisol levels as markers ofchronic stress among post-menopausal women. Along with the above findings, prevalence of these psychosomatic disorders among the study group and the variation in the levels salivary cortisol also has been analysed.

In this study, correlation of salivary cortisol has been done with various psychosomatic disorders while mean values and standard deviation for each psychosomatic disorders prevalent in the head and neck region of the study group, enlisted in the inclusion criteria have been provided (Table 4).

Significant increase in levels of salivary cortisol was noticed in the study groups. Head and neck psychosomatic disorders like TMD's, MPDS, Apthous stomatitis, Atypical facial pain, Oral lichen planus(OLP), Burning mouth syndrome [BMS]/ Glossopyrosis were seen primarily among our subjects. These findings manifested singularly or in combination along with other findings like Dysgeusia, Xerostomia, Attrition and Halitosis.

After menopause, adrenal steroid secretion provides a low level of estradiol, in response to adrenocorticotropic hormone (ACTH) secretion. Cortisol is also released under the influence of hypophysial ACTH.^17^ This study evaluates the relationship of cortisol level in unstimulated whole saliva with severity of psychosomatic disorders among post-menopausal women. In our study we found that there was significant increase of salivary cortisol levels among the study groups and also the levels increased with the post-menopause duration.

In our study mean value of salivary cortisol was seen to be highest among Apthous stomatitis (37.97 ng/ml) and least among subjects who experienced halitosis (23.33 ng/ml). Hence this study directly projects association of salivary cortisol levels to various psychosomatic disorders of the head and neck region among the post-menopausal women. Hence this association implies that salivary cortisol may be used as an important biomarker of stress. The present study is a rarity and first of its kind as such association and correlation between psychosomatic disorders and salivary cortisol levels among the post-menopausal women has not been conducted.

Further research in this field has ample scope to explore various types of stressors, factors that cause stress which lead to manifestation of psychosomatic disorders. Also research activities directed towards various approaches to management of these patients, so as to minimise the sufferings of the elderly women should be encouraged. The dental practitioners are recommended to thoroughly examine and counsel each patient before and after the commencement of treatment in post-menopausal/geriatric women.

## Conclusion

Stress is a fact of everyday life. When people reach out for help, they are often dealing with situations, circumstances and stressors in their lives that leave them feeling physically and emotionally overwhelmed. Many people especially geriatric patients feel that they have very little skills or resources to deal with the high levels of stress that they are experiencing. The results of this study recommend that stress evaluation could be done on a regular basis for post- menopausal women. For those individuals who do not reveal about their psychological distress, analysis of salivary cortisol will aid in the diagnosis of their situation. Also stomatologists need to thoroughly examine the head and neck for stress related disorders so that the management and relief can be delivered to the ailing patient.
